# Functional neural correlates of psychopathy: a meta-analysis of MRI data

**DOI:** 10.1038/s41398-020-0816-8

**Published:** 2020-05-06

**Authors:** Philip Deming, Michael Koenigs

**Affiliations:** 1 Department of Psychology, University of Wisconsin-Madison, 1202 West Johnson St., Madison, Wisconsin 53706 USA; 2 Department of Psychiatry, University of Wisconsin-Madison, 6001 Research Park Blvd., Madison, Wisconsin 53719 USA

**Keywords:** Biomarkers, Psychiatric disorders

## Abstract

Neuroimaging studies over the last two decades have begun to specify the neurobiological correlates of psychopathy, a personality disorder that is strongly related to criminal offending and recidivism. Despite the accumulation of neuroimaging studies of psychopathy, a clear and comprehensive picture of the disorder’s neural correlates has yet to emerge. The current study is a meta-analysis of functional MRI studies of psychopathy. Multilevel kernel density analysis was used to identify consistent findings across 25 studies (460 foci) of task-related brain activity. Psychopathy was associated with increased task-related activity predominantly in midline cortical regions overlapping with the default mode network (dorsomedial prefrontal cortex, posterior cingulate, and precuneus) as well as medial temporal lobe (including amygdala). Psychopathy was related to decreased task-related activity in a region of the dorsal anterior cingulate cortex overlapping with the salience network. These findings challenge predominant theories of amygdala hypoactivity and highlight the potential role of hyperactivity in medial default mode network regions and hypoactivity in a key node of the salience network during task performance in psychopathy.

## Introduction

Two decades of neuroimaging studies have begun to specify the neurobiological correlates of psychopathy, a personality disorder characterized by callous and impulsive antisocial behavior. Present in approximately one-quarter of adult male prison inmates^1^, psychopathy is a significant predictor of violent reoffending^2^ that costs the United States an estimated $460 billion per year^3^, making psychopathy one of the most costly mental health disorders. Despite the accumulation of neuroimaging studies of psychopathy, a clear and comprehensive picture of the disorder’s neural correlates has yet to emerge.

Specifying the neurobiological correlates of psychopathy could have implications for the diagnosis and treatment of the disorder, as well as for the criminal justice system. Clinically, the identification of neural correlates of psychopathy could inform the development of biomarkers for vulnerability and early detection, anatomical targets for intervention, and predictors of treatment response. Furthermore, given the relatively high prevalence of psychopathy among criminal offenders and its predictive utility for violent reoffending, there is growing interest in the relevance of neuroimaging data for predicting future offense^4^, as well as for influencing sentencing of psychopathic offenders^5^. Hence, neuroimaging research on psychopathy has multiple potential applications.

Neuroimaging studies to date have generally tested specific theoretical models of neurobiological dysfunction in psychopathy. In fact, we identified 68 neuroimaging studies of psychopathy that used hypothesis-driven, region-of-interest (ROI) analyses, compared with 50 studies that used whole-brain analyses. The amygdala, in particular, is the focus of a robust literature on impaired fear and threat processing in psychopathy^6,7^. Psychopathic individuals show diminished amygdala activity during aversive conditioning^8^, facial emotion recognition^9,10^, and moral judgment^11,12^. Amygdala volume reductions^13,14^ and surface deformations^15^ have also been observed in psychopathic individuals. A region densely interconnected with the amygdala, the ventromedial prefrontal cortex (vmPFC), has also received substantial attention in the psychopathy literature^16,17^. Psychopathic individuals show reduced vmPFC activity during moral judgment^18^ and reduced vmPFC volume^19,20^. Amygdala and vmPFC have been identified by one theoretical perspective as the primary circuit of dysfunction in psychopathy^21^. According to this perspective, deficits in these regions underlie psychopathic individuals’ failure to pair negative and positive outcomes with their own actions.

Another perspective expands the scope of neural dysfunction beyond amygdala and vmPFC to the paralimbic cortex more broadly, including anterior cingulate cortex (ACC), posterior cingulate cortex (PCC), temporal pole, insula, and parahippocampal gyrus^22^. Indeed, psychopathy has been associated with structural^19,23,24^ and functional^25–27^ abnormalities in these regions. Moreover, deficits similar to those of psychopathic individuals have been observed in patients with lesions in paralimbic regions, including vmPFC^28–30^, ACC^31,32^, and amygdala^33^.

Psychopathic individuals also display heightened sensitivity to rewards and sensation-seeking behavior^34^, which has been linked to increased volume and activity in the striatum^35^. Psychopathic individuals show enlargements in several subregions of the striatum, including caudate, putamen, and nucleus accumbens^35,36^, and increased activity of nucleus accumbens during reward anticipation^37^. Increased volume and activity in the striatum may thus contribute to psychopathic individuals’ impulsive, sensation-seeking behavior. Although the studies outlined above highlight the importance of striatum and paralimbic regions—particularly amygdala and vmPFC—in psychopathy, reliance on primarily hypothesis-driven analyses may limit the identification of other relevant brain circuits.

A recent meta-analysis of fMRI studies aimed to specify the neural correlates of psychopathy using a whole-brain approach^38^. Psychopathy was negatively related to task-based activity in bilateral dorsolateral prefrontal cortex (dlPFC), left dorsomedial prefrontal cortex (dmPFC), and right amygdala, and positively related to activity in bilateral anterior insula. However, methodological characteristics of this recent meta-analysis limit the interpretations that can be drawn from these findings. First, the prior study included the effects of the distinct subsets of psychopathic traits in their meta-analysis of total psychopathy. Conflating subsets of psychopathic traits with the disorder itself could lead to the interpretation that a particular brain region or network is related to psychopathy overall, when in fact it is related to a restricted subset of traits. In addition, their meta-analytic method, activation likelihood estimate (ALE), uses coordinates of individual activation peaks as the unit of analysis, thereby potentially allowing a small number of studies reporting relatively high numbers of activation coordinates to drive the meta-analytic results^39,40^. By contrast, the current state-of-the-art meta-analytic approach, multilevel kernel density analysis (MKDA), uses the contrast map from an individual study as the unit of analysis and weighs study quality and sample size, allowing for greater generalization of findings^39,40^.

Thus, the purpose of the current study is to use MKDA to identify consistent relationships between psychopathy and neural activity across a variety of tasks. Based on the previous findings reviewed above, psychopathy was predicted to be related to decreased activity in amygdala, vmPFC, ACC, PCC, and insula, and to increased activity in the striatum. The current study originally had three additional goals: (1) to identify structural neural correlates of psychopathy, (2) to identify neural correlates of two well-established subsets of psychopathic traits, and (3) to determine whether dysfunctional neural activity in psychopathy varies by task. However, an insufficient number of studies contributed to each of the models addressing these three goals. We present preliminary results from these models in the Supplementary Materials (Tables S1–S5), and focus our discussion on the functional neural correlates of psychopathy across tasks.

## Methods

### Study selection and coding

**Fig. 1:**
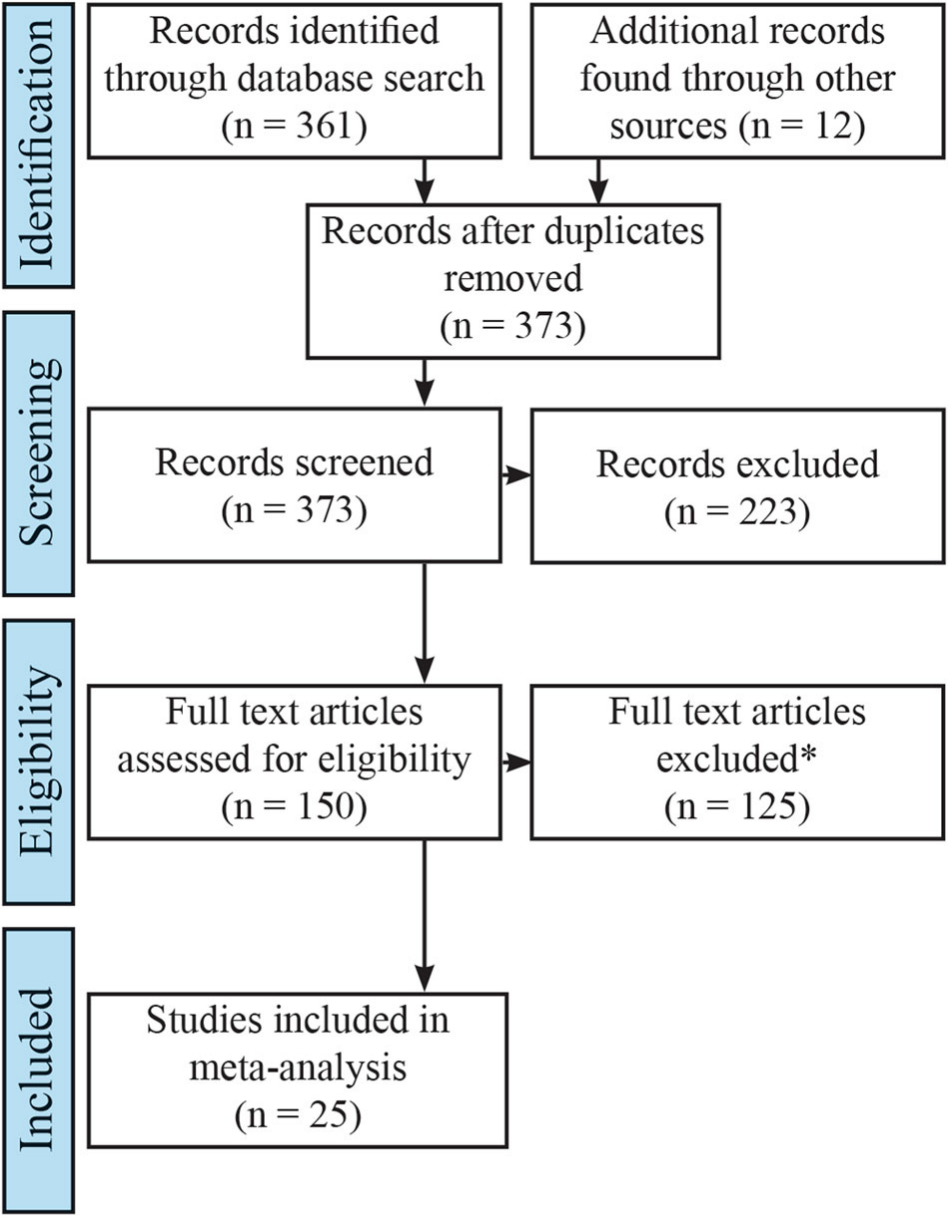
Flowchart of the literature search process. After a full-text review, studies were excluded for using region of interest (ROI) rather than whole-brain analyses (68 studies), examining neural measures other than task-based neural activity (21 studies), summarizing prior literature rather than reporting new findings (18 studies), not reporting peak coordinates of findings related to total psychopathy (15 studies), grouping impulsive, non-psychopathic participants with psychopathic participants (1 study), excluding non-psychopathic participants from a continuous analysis of psychopathy (1 study), and reporting single-case results (1 study).

We conducted a forward literature search in PubMed, PsycINFO, and Google Scholar, and a backward literature search in the reference sections of relevant papers. The following search terms were used in the forward literature search: psychopathy, psychopathic, magnetic resonance imaging, MRI, functional magnetic resonance imaging, fMRI, and neuroimaging. Studies published prior to December 31, 2019 were included based on the following criteria: (1) reported peak coordinates in Talairach^41^ or Montreal Neurological Institute (MNI) template space, (2) used whole-brain analyses, (3) sampled only participants aged 18 and older, (4) reported group statistics rather than single-case results, and (5) compared brain activity with a measure of psychopathy. The results from group comparisons (e.g., psychopaths vs. controls) and continuous analyses (i.e., variability across a range of psychopathy scores) were included. The resulting dataset included 460 foci from 87 contrasts in 25 studies of task-based functional activity (Table 1). Figure 1 displays the number of studies excluded at each stage of the literature search.

**Table 1:** Summary of studies included in the functional meta-analysis for total psychopathy.

				No. of foci	
Study	*N*	Psychopathy measure	Task	−	+
Abe et al.^70^	67	PCL-R	Dishonest decision-making	3	0
Contreras-Rodríguez et al.^71^	44	PCL-R	Facial emotion processing	0	9
Cope et al.^72^	137	PCL-R	Viewing drug-related images	10	1
Decety et al.^73^	155	PCL-R	Empathy for harmed person	8	24
Deeley et al.^42^	15	PCL-R	Facial emotion processing	15	0
Fede et al.^74^	245	PCL-R	Moral judgment	4	0
Gregory et al.^75^	50	PCL-R	Reversal learning	6	16
Harenski et al.^12^	157	PCL-R	Moral judgment	9	0
Harenski et al.^18^	32	PCL-R	Moral judgment	4	1
Kiehl et al.^76^	16	PCL-R	Abstract word processing	1	0
Larson et al.^77^	49	PCL-R	Fear-potentiated startle	1	3
Marsh and Cardinale^11^	33	PPI-R	Moral judgment	4	1
Meffert et al.^78^	46	PCL-R	Viewing interacting hands	76	33
Mier et al.^79^	29	PCL-R	Facial emotion processing	9	2
Müller et al.^80^	12	PCL-R	Viewing emotional images	11	25
Osumi et al.^62^	20	PSPS	Economic decision-making	6	0
Pujol et al.^56^	44	PCL-R	Moral judgment	5	1
Rilling et al.^81^	30	PSPS	Economic decision-making	23	12
Rodman et al.^82^	46	PCL-R	Inhibitory self-control	0	6
Schultz et al.^83^	50	PCL-R	Fear conditioning	0	14
Sethi et al.^84^	232	SRP-SF	Facial emotion processing	24	0
Shane and Groat^85^	67	PCL-R	Emotion regulation	18	55
Shao and Lee^86^	48	PPI-R	Instructed lying	3	0
Sommer et al.^87^	28	PCL-R	Mentalizing	0	3
Yoder et al.^88^	88	PCL-R	Moral judgment	14	0
Total no. of foci				254	206
Total no. of studies				21	16

*PCL-R* psychopathy checklist-revised^1^; *PPI-R* psychopathic personality inventory-revised^89^; *PSPS* primary and secondary psychopathy scales^90^; *SRP-SF* self-report psychopathy short form^91^.

We ensured that included contrasts were coded such that “positive” activation indicated greater activity associated with the cognitive function of interest in each study. For example, contrasts from studies examining facial emotion processing were coded as fear faces > neutral faces^42^, and contrasts from studies examining moral judgment were coded as moral > non-moral^12^. Each reported coordinate was coded as relating positively or negatively to total psychopathy. Accuracy of the foci and corresponding information was verified by a second coder.

We determined that models with at least 15 included studies would achieve sufficient power in MKDA. Although no published standards exist for the number of included studies required for MKDA, there is evidence that 17–20 experiments are sufficient for another meta-analytic approach (ALE)^39,43^. Given the statistical advantages of MKDA, we used a threshold of 15 studies. See Tables S1–S5 for preliminary results from models that did not meet this threshold.

### Multilevel kernel density analysis

Analyses were conducted using the MKDA toolbox in SPM12^39^. Foci in Talairach space were converted to MNI space^44^. Binary indicator maps for each contrast were created by convolving peak foci using a 10-mm spherical kernel, with voxels related to psychopathy assigned a value of 1. Next, each indicator map was weighted by the square root of the study’s sample size, and the weighted average of the indicator maps was computed. The resulting density maps represented the proportion of studies showing psychopathy relationships within 10 mm. Finally, statistical thresholding was determined via 5,000 Monte Carlo simulations. The results were considered significant at *p*_*FWE*_ < 0.05 using height-based criteria or cluster extent-based criteria. Images for the figures were generated in AFNI (16.0)^45^. Individual models were run for positive and negative relationships with total psychopathy.

## Results

**Fig. 2:**
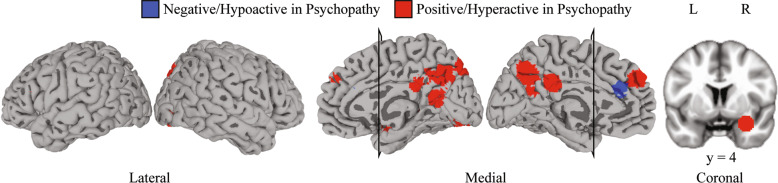
Consistent relationships between total psychopathy scores and brain function during experimental tasks. Medial views depict the location of the coronal slice.

Across all functional studies, total psychopathy was negatively related to neural activity in dorsal ACC (Fig. 2 and Table 2). In contrast, total psychopathy was positively related to neural activity in a large, bilateral portion of medial parietal and occipital cortex (including PCC and precuneus), bilateral dmPFC, right inferior frontal gyrus, right posterior orbitofrontal cortex, right medial temporal cortex (including amygdala), right hippocampus, and right parahippocampal gyrus (Fig. 2 and Table 2).

**Table 2:** Significant associations between brain function and psychopathy.

Region(s)	Hemi.	Direction	Peak MNI coordinates (*x*, *y*, *z*)	Size (voxels)	Threshold
Anterior cingulate	L	−	−6, 32, 22	293	Extent
Dorsomedial prefrontal cortex	L	+	−2, 56, 28	1	Height
Dorsomedial prefrontal cortex	L/R	+	−2, 50, 34	347	Extent
Dorsomedial prefrontal cortex	L/R	+	2, 44, 32	69	Height
Inferior frontal gyrus (pars orbitalis)	R	+	40, 40, −8	301	Extent
Posterior orbitofrontal cortex	R	+	26, 8, −16	12	Height
Amygdala/temporal pole	R	+	30, 4, −20	399	Extent
Hippocampus/parahippocampal gyrus	R	+	24, −36, 2	105	Extent
Hippocampus/parahippocampal gyrus	R	+	30, −40, −4	337	Height
Cerebellum/cuneus/inferior occipital cortex/posterior cingulate/precuneus/superior occipital cortex	L/R	+	12, −70, 16	4272	Extent

## Discussion

Through a meta-analysis of functional neuroimaging studies of psychopathy, we identified several reliable neural correlates of psychopathy. We discuss three main conclusions from these results. For two of the main conclusions, we draw on a body of literature that has identified three intrinsic, large-scale networks that serve core cognitive functions^46–51^: the default mode network (DMN), salience network (SN), and frontoparietal network (FPN). Whereas DMN increases activity during self-referential processing and decreases activity during externally focused, non-self-referential tasks^52^, FPN increases activity during cognitively demanding, externally focused tasks^48,49^. There is evidence that SN, which is particularly important for detecting salient external stimuli, is responsible for switching between the two anticorrelated networks, DMN and FPN^48,49,51^. Our findings suggest that psychopathy is related to dysfunction within DMN and SN across a variety of tasks.

**Fig. 3:**
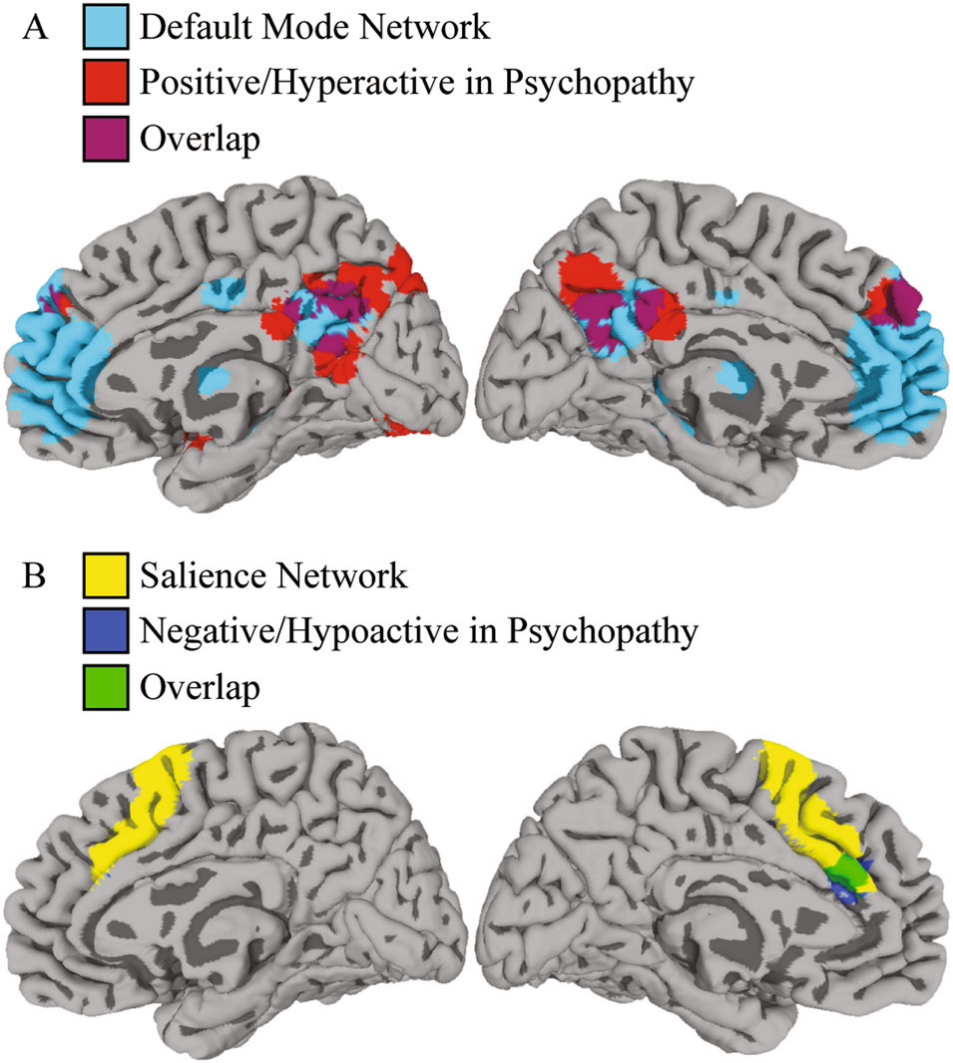
Large-scale networks and regions associated with psychopathy. **a** Overlap between default mode network (DMN) and regions of hyperactivity in psychopathy across a variety of tasks. **b** Overlap between salience network (SN) and regions of hypoactivity in psychopathy across a variety of tasks. A functional connectivity-based atlas was used to display canonical DMN and SN regions^92^.

First, we note the considerable overlap between the areas of psychopathy-related positive activity and the DMN, particularly in dmPFC and PCC/precuneus (Fig. 3a). One interpretation, previously outlined by Freeman et al.^53^, is that psychopathic individuals fail to deactivate these midline DMN regions during externally focused tasks. Such a failure could result in increased competition between DMN and externally oriented attention networks (such as FPN), disrupt the shift of attention to the external task, and lead to corresponding performance deficits^54^. However, because the current meta-analytic evidence does not directly corroborate this interpretation, future studies are warranted to clarify whether overactivity within midline DMN regions in psychopathy reflects less task-associated deactivation or greater task-associated activation. Findings of reduced deactivation in DMN regions may align with theories that highlight attention deficits in psychopathy^55^, although future studies could investigate whether dysfunction in networks involved in allocating attention to cognitively demanding tasks, such as FPN or SN, is more closely associated with psychopathic individuals’ attention deficits. In psychopathy, PCC/precuneus is not only overactive across a variety of tasks, but also less functionally and structurally connected to other DMN regions, including dmPFC^56,57^ and vmPFC^58^, and to a region of FPN^25^.

Second, as predicted, psychopathy was related to reduced dorsal ACC activity across a variety of tasks. Research in healthy humans and animal populations has implicated the dorsal ACC in a number of processes, including experiencing negative affect and pain, cognitive control^59^, and linking task context to strategy^60^. As a key SN node (Fig. 3b), the dorsal ACC, along with insula, is neuroanatomically situated to send rapid signals deactivating DMN and activating FPN^48,49,51^. Indeed, there is evidence that SN functions at the top of a hierarchy that includes DMN and FPN, modulating the two anticorrelated networks via inhibitory connections to DMN and excitatory connections to FPN^47^. Thus, one interpretation of reduced dorsal ACC activity in psychopathy, in combination with increased DMN activity, is that this SN node fails to properly deactivate DMN, leading to competition between attentional resources while psychopathic individuals are engaged in an externally oriented task. While speculative, the hypothesis of reduced SN influence on DMN activity could be tested via methods such as Granger causality^49^ or dynamic causal modeling^48^. Prior analyses of resting-state functional connectivity lend initial corroborating evidence of altered dynamics between the three networks: dorsal ACC (of the SN) and precuneus (of the DMN) are less functionally connected to the right intraparietal sulcus (of the FPN) in psychopathic offenders^25^.

Notably, some researchers have argued that common dysfunction within and between these intrinsic networks (DMN, FPN, and SN) underlies a range of psychopathology^46,50^. For example, a diminished modulating effect of SN on DMN and FPN has been observed in patients with schizophrenia^61^ and elderly individuals with mild cognitive impairment^51^. However, disorders may be distinguished by unique dysfunction in specific nodes of these core networks (e.g., hyperactive subgenual ACC in depression)^46^. Further inquiry is necessary to establish whether hyperactivity in medial DMN nodes and underactivity in SN is unique to psychopathy or shared among other disorders.

Third, we found overall increased levels of amygdala activity associated with psychopathy. Contrary to previous ROI-based studies, we observed no evidence of decreased amygdala activity across a variety of tasks. (Even preliminary analyses of socioemotional processing, e.g., facial emotion processing in Table S4, failed to yield evidence of decreased amygdala activity.) This finding challenges predominant views in the field, which identify amygdala hypoactivity as a key neural deficit in psychopathy^8,11,15,21,22,62,63^. Further research is thus necessary to more thoroughly characterize amygdala dysfunction in psychopathy, and the following considerations may guide those studies. First, the amygdala is a heterogeneous structure, with distinct subnuclei that subserve different functions and interact with different brain networks^64^. Psychopathy may be linked to hyperactivity in specific amygdala subnuclei and hypoactivity in others, a possibility that has already been raised by one theoretical perspective^65^. Indeed, several studies have found that amygdala relationships with psychopathy vary by subnucleus^14,66^. Second, an emerging view proposes that, in healthy individuals, the amygdala may switch its affective mode (i.e., positive or negative, appetitive or avoidant) across situations^67^. This raises the possibility that amygdala dysfunction in psychopathy varies by context. At a minimum, the present results suggest that amygdala dysfunction in psychopathy is more complex than simply reduced activation resulting in diminished fear conditioning. Future studies should examine whether altered modules (i.e., amygdala subnuclei) or modes account for this complexity.

No findings from the previous neuroimaging meta-analysis of psychopathy were replicated by the current meta-analysis^38^. Prior findings of consistent hyperactivation in the fronto-insular cortex and hypoactivation in bilateral dlPFC, dmPFC, and right amygdala were not corroborated by our models. In fact, two findings diametrically oppose those of the previous meta-analysis: dmPFC and right amygdala were hyperactive in the current meta-analysis, but hypoactive in the previous study. Though unexpected, the differences in results could be at least partly attributed to methodological differences (i.e., study inclusion criteria, contrast selections, and meta-analytic methods), as detailed in the introduction of this paper.

A primary limitation of the present meta-analysis is the exclusion of studies that employed ROI analyses, necessitated by MKDA’s assumption that all included studies explored the same space (in this case, the whole brain). As a result, the present meta-analytic results do not represent the whole of the neuroimaging literature on psychopathy. This limitation could be overcome through the use of image-based meta-analysis, the “gold standard” of neuroimaging meta-analytic methods^68^. Image-based meta-analysis requires the compilation of full statistical maps from each study to assess the overall effect sizes at each voxel. Although this method would present significant challenges, its results would offer a more complete picture of the underlying neurobiology of psychopathy. Further, the meta-analysis did not control for potential sample overlap between studies, nor did it consider separately the results from categorical or continuous analyses. While this meta-analysis included studies of community samples, which may feature relatively low levels of psychopathy, the results were largely unchanged when excluding these studies (Table S6). Last, the present results offer insights into aberrant neural activity, but no evidence regarding connectivity within networks, or how information is represented within specific brain regions in psychopathy. Previous studies have investigated the former^25,26,58,69^, and machine-learning studies could address the latter.

In sum, these results associate psychopathy with neural abnormalities concentrated primarily in medial prefrontal, parietal, and temporal cortices. Crucially, no single current neurobiological theory of psychopathy accounts for the observed functional abnormalities, which include multiple cortical and subcortical regions. Consistent amygdala hyperactivity, observed here, challenges predominant views in the field that amygdala hypoactivity underlies psychopathy. A more complex characterization of amygdala function in psychopathy is necessary to account for previously observed hypoactivity and currently observed hyperactivity. Moreover, the present results highlight two intrinsic networks in which task-based activity is altered in psychopathy. Characterizing the dynamic interactions of these networks in future studies could potentially further illuminate a core neurobiological deficit of the disorder. The present meta-analysis thus provides novel insight into the neural correlates of psychopathy.

## Supplementary information

Supplementary Materials

## Data Availability

Matlab code for the MKDA toolbox is publicly available at https://github.com/canlab/Canlab_MKDA_MetaAnalysis.
