# Supplementary material for: Functional neural correlates of psychopathy: a meta-analysis of MRI data
Source: Transl Psychiatry. 2020 May 6;10:133. doi: 10.1038/s41398-020-0816-8 (PMC7203015; doi:10.1038/s41398-020-0816-8)
Supplement: Supplementary file 1 — Supplementary Materials [file 41398_2020_816_MOESM1_ESM.docx]

**SUPPLEMENTARY METHODS**

Here we report preliminary analyses based on three original study aims: 1) to identify consistent relationships between psychopathy and gray matter structure, 2) to identify the functional and structural neural correlates of two distinct clusters of psychopathic traits (interpersonal/affective traits and impulsive lifestyle/antisocial traits), and 3) to determine whether functional neural correlates of psychopathy vary by task. Each of these preliminary models was excluded from the main text because they failed to meet the threshold of 15 included studies. We also report follow-up analyses that assessed whether the neural correlates of psychopathy differed when studies of community samples were excluded.

All of the studies included in the preliminary analyses described above were identified using the methods and search terms described in the manuscript. Twelve studies of gray matter structure in psychopathy were determined to meet the inclusion criteria (**Table S1**). For each structural study, the gray matter measure (e.g., gray matter volume, cortical thickness) was coded. Analyses were first collapsed across all available measures of gray matter structure (“All Structural”), then restricted to studies of gray matter volume (see **Table S2** for results).

For studies of gray matter structure and neural activity, we conducted separate meta-analyses for the two well-established subsets of psychopathic traits to determine whether these traits have distinct neural correlates. Studies contributing foci to models for each of the subsets of traits are reported in **Table S1 and Table S3**, and results are reported in **Table S2 and Table S4**.

To determine whether functional neural correlates of psychopathy vary by task, we separately examined tasks involving facial emotion processing (i.e., stimuli were faces expressing emotion; tasks were emotion identification/matching, gender identification, or passive viewing), empathy (i.e., stimuli were images of two individuals interacting in either harmful or prosocial ways; tasks were emotion recognition, perspective-taking, or passive viewing), and moral judgment (i.e., stimuli were visual or written depictions of moral scenarios; tasks were moral violation identification/rating or passive viewing). Studies contributing to each task category can be found in **Table S3**, and results are in **Table S4**.

To ensure that the meta-analytic findings were not primarily driven by studies of community samples, which may reflect subclinical variation in psychopathic traits among a healthy population rather than the disorder of psychopathy itself, we ran additional models of total psychopathy excluding studies of community samples. **Table S5** shows consistent relationships between total psychopathy and gray matter structure (8 studies, 75 foci) excluding studies of community samples, while **Table S6** shows consistent relationships between total psychopathy and task-related activity (20 studies, 388 foci) excluding studies of community samples.

**SUPPLEMENTARY TABLES**

| **Table S1**. Summary of studies included in the structural meta-analysis | | | | | | | | | | | | |
| --- | --- | --- | --- | --- | --- | --- | --- | --- | --- | --- | --- | --- |
| **Study** | **N** | **Psychopathy Measure** | **Gray Matter Measure** | **Total Psychopathy Foci** | | **Interpersonal/ Affective Foci** | | | **Impulsive Lifestyle/ Antisocial Foci** | | |  |
|  |  |  |  | **-** | **+** | **-** | **+** | **-** | | **+** |  |  |
| Beckwith et al. (2018) | 155 | PPI | BVC | 3 | 3 | 0 | 0 | 0 | | 0 |  |  |
| Contreras-Rodríguez et al. (2015) | 44 | PCL-R | GMV, GMC | 19 | 0 | 14 | 0 | 0 | | 13 |  |  |
| Cope et al. (2012) | 66 | PCL-R | GMV | 2 | 2 | 0 | 2 | 7 | | 2 |  |  |
| de Oliveira-Souza et al. (2008) | 30 | PCL-SV | GMV | 22 | 0 | 0 | 0 | 0 | | 0 |  |  |
| Gregory et al. (2012) | 66 | PCL-R | GMV | 29 | 0 | 0 | 0 | 0 | | 0 |  |  |
| Kolla et al. (2014) | 37 | PCL-R | GMV | 2 | 0 | 0 | 0 | 0 | | 0 |  |  |
| Korponay et al. (2017) | 124 | PCL-R | GMV | 0 | 0 | 0 | 0 | 0 | | 6 |  |  |
| Ly et al. (2012) | 52 | PCL-R | CT | 13 | 0 | 0 | 0 | 0 | | 0 |  |  |
| Miskovich et al. (2018) | 716 | PCL-R | CG | 2 | 0 | 1 | 2 | 1 | | 0 |  |  |
| Müller et al. (2008) | 34 | PCL-R | GMV | 1 | 0 | 0 | 0 | 0 | | 0 |  |  |
| Schiffer et al. (2011)* | 51 | PCL-SV | GMV | 1 | 4 | 1 | 2 | 1 | | 2 |  |  |
| Vieira et al. (2014) | 35 | TriPM | GMV | 1 | 0 | 0 | 0 | 0 | | 0 |  |  |
| Total No. Foci |  |  |  | 95 | 9 | 16 | 6 | 9 | | 23 |  |  |
| Total No. Studies |  |  |  | 11 | 3 | 3 | 3 | 3 | | 4 |  |  |
| PCL-R = Psychopathy Checklist-Revised (1); PCL-SV = Psychopathy Checklist-Screening Version (83); PPI = Psychopathic Personality Inventory (84); TriPM = Triarchic Psychopathy Measure (85)  BVC = Brain Volume Changes; GMV = Gray Matter Volume; GMC = Gray Matter Concentration; CT = Cortical Thickness; CG = Cortical Gyrification  *Of the five studies that reported findings for the two clusters of psychopathic traits (interpersonal/affective and impulsive lifestyle/antisocial), one study controlled for the other cluster of traits in statistical models. | | | | | | | | | | | | |

| **Table S2**. Significant associations between brain structure and psychopathy | | | | | | |
| --- | --- | --- | --- | --- | --- | --- |
| **Measure** | **Region(s)** | **Hemi.** | **Direction** | **Peak MNI Coordinates**  **(x, y, z)** | **Size (Voxels)** | **Threshold** |
| **Total Psychopathy** | | | | | | |
| All Structural | Dorsomedial Prefrontal Cortex/  Middle Frontal Gyrus/  Rostral Anterior Cingulate/ Superior Frontal Gyrus | L | - | -14, 54, 24 | 978 | Extent |
|  | Fusiform Gyrus/  Inferior Temporal Gyrus/  Medial Temporal Pole | R | - | 36, 10, -38 | 1020 | Extent |
|  | Fusiform Gyrus/  Inferior Temporal Gyrus | L | - | -38, -4, -40 | 927 | Extent |
|  | Caudate/  Nucleus Accumbens/  Subgenual Anterior Cingulate | L/R | + | -4, 20, -2 | 1371 | Extent |
| GMV | Dorsomedial Prefrontal Cortex/  Superior Frontal Gyrus | L | - | -12, 58, 20 | 674 | Extent |
|  | Dorsomedial Prefrontal Cortex/  Superior Frontal Gyrus | R | - | 12, 44, 38 | 757 | Extent |
|  | Fusiform Gyrus/  Inferior Temporal Gyrus/  Medial Temporal Pole | L | - | -36, -4, -40 | 759 | Extent |
|  | Caudate | L | + | -10, 18, -2 | 112 | Height |
| **Interpersonal/Affective Psychopathic Traits** | | | | | | |
| All Structural | Midcingulate | L/R | - | 4, -4, 34 | 493 | Extent |
|  | *No significant clusters* |  | + |  |  |  |
| GMV | Middle Frontal Gyrus | R | - | 42, 48, 12 | 430 | Height |
|  | Middle Frontal Gyrus | L | - | -34, 42, 32 | 509 | Height |
|  | Dorsomedial Prefrontal Cortex | R | - | 8, 42, 46 | 506 | Height |
|  | Inferior Frontal Gyrus (Tri., Orb.) | R | - | 52, 34, -2 | 376 | Height |
|  | Temporal Pole | L | - | -48, 16, -26 | 485 | Height |
|  | Amygdala/  Anterior Insula/  Posterior Orbitofrontal Cortex/  Putamen/  Temporal Pole | R | - | 24, 6, -14 | 515 | Height |
|  | Amygdala/  Hippocampus/  Posterior Orbitofrontal Cortex/  Putamen | L | - | -20, 0, -14 | 515 | Height |
|  | Cerebellum | L | - | -18, -64, -22 | 515 | Height |
|  | Frontal Pole/  Middle Frontal Gyrus | L | + | -22, 58, 14 | 894 | Height |
|  | Superior Frontal Gyrus | L | + | -16, 46, 18 | 4 | Height |
| **Impulsive Lifestyle/Antisocial Psychopathic Traits** | | | | | | |
| All Structural | Fusiform Gyrus | R | - | 40, -38, -24 | 511 | Height |
|  | Caudate | L | + | -16, 20, 2 | 248 | Height |
| GMV | Fusiform Gyrus/  Hippocampus/  Inferior Temporal Gyrus /  Middle Temporal Gyrus/  Superior Temporal Gyrus | L | - | -52, -16, -18 | 2510 | Height |
|  | Middle Temporal Gyrus | L | - | -58, -40, -6 | 509 | Height |
|  | Caudate | L | + | -16, 20, 2 | 248 | Height |
| GMV = Gray Matter Volume  Inferior Frontal Gyrus subregions are labeled as Op. = pars Opercularis, Orb. = pars Orbitalis, Tri. = pars Triangularis. | | | | | | |

| **Table S3**. Summary of studies included in the functional meta-analysis | | | | | | | | | |
| --- | --- | --- | --- | --- | --- | --- | --- | --- | --- |
| **Study** | **N** | **Psychopathy Measure** | **Task Category** | **Total Psychopathy Foci** | | **Interpersonal/ Affective Foci** | | **Impulsive Lifestyle/ Antisocial Foci** | |
|  |  |  |  | **-** | **+** | **-** | **+** | **-** | **+** |
| Abe et al. (2018) | 67 | PCL-R | NC | 3 | 0 | 0 | 0 | 0 | 0 |
| Anderson et al. (2018)***** | 168 | PCL-R | NC | 0 | 0 | 30 | 0 | 0 | 0 |
| Caldwell et al. (2015) | 316 | PCL-R | NC | 0 | 0 | 2 | 0 | 0 | 0 |
| Contreras-Rodríguez et al. (2014) | 44 | PCL-R | FEP | 0 | 9 | 0 | 3 | 5 | 0 |
| Cope et al. (2014)***** | 137 | PCL-R | NC | 10 | 1 | 0 | 6 | 46 | 0 |
| Decety et al. (2015) | 155 | PCL-R | E | 8 | 24 | 8 | 4 | 0 | 0 |
| Deeley et al. (2006) | 15 | PCL-R | FEP | 15 | 0 | 0 | 0 | 0 | 0 |
| Deming et al. (2018)***** | 57 | PCL-R | NC | 0 | 0 | 0 | 0 | 4 | 0 |
| Fede et al. (2016) | 245 | PCL-R | MJ | 4 | 0 | 0 | 0 | 0 | 0 |
| Gregory et al. (2015) | 50 | PCL-R | NC | 6 | 16 | 0 | 0 | 0 | 0 |
| Han et al. (2012) | 32 | PPI-R | FEP | 0 | 0 | 10 | 1 | 0 | 0 |
| Harenski et al. (2014) | 157 | PCL-R | MJ | 9 | 0 | 1 | 0 | 4 | 0 |
| Harenski et al. (2010) | 32 | PCL-R | MJ | 4 | 1 | 0 | 0 | 0 | 0 |
| Kiehl et al. (2004) | 16 | PCL-R | NC | 1 | 0 | 0 | 0 | 0 | 0 |
| Larson et al. (2013) | 49 | PCL-R | NC | 1 | 3 | 0 | 0 | 0 | 0 |
| Marsh & Cardinale (2014) | 33 | PPI-R | MJ | 4 | 1 | 0 | 0 | 0 | 0 |
| Meffert et al. (2013) | 46 | PCL-R | E | 76 | 33 | 0 | 0 | 0 | 0 |
| Mier et al. (2014) | 29 | PCL-R | FEP | 9 | 2 | 0 | 0 | 0 | 0 |
| Müller et al. (2003) | 12 | PCL-R | NC | 11 | 25 | 0 | 0 | 0 | 0 |
| Osumi et al. (2012) | 20 | PSPS | NC | 6 | 0 | 0 | 0 | 0 | 0 |
| Pujol et al. (2012) | 44 | PCL-R | MJ | 5 | 1 | 0 | 0 | 0 | 0 |
| Rilling et al. (2007) | 30 | PSPS | NC | 23 | 12 | 0 | 0 | 0 | 0 |
| Rodman et al. (2016) | 46 | PCL-R | NC | 0 | 6 | 0 | 0 | 0 | 0 |
| Sadeh et al. (2013)***** | 49 | NEO-FFI | NC | 0 | 0 | 0 | 2 | 0 | 10 |
| Schultz et al. (2016) | 50 | PCL-R | NC | 0 | 14 | 0 | 0 | 0 | 0 |
| Sethi et al. (2018) | 232 | SRP-SF | FEP | 24 | 0 | 0 | 0 | 0 | 0 |
| Shane & Groat (2018) | 67 | PCL-R | NC | 18 | 55 | 0 | 0 | 0 | 0 |
| Shao & Lee (2017) | 48 | PPI-R | NC | 3 | 0 | 0 | 0 | 0 | 0 |
| Sommer et al. (2010) | 28 | PCL-R | NC | 0 | 3 | 0 | 0 | 0 | 0 |
| Yoder, Harenski et al. (2015) | 88 | PCL-R | MJ | 14 | 0 | 6 | 6 | 13 | 5 |
| Yoder, Porges et al. (2015)***** | 43 | PPI-R | NC | 0 | 0 | 5 | 1 | 0 | 2 |
| Total No. Foci |  |  |  | 254 | 206 | 62 | 23 | 72 | 17 |
| Total No. Studies |  |  |  | 21 | 16 | 7 | 7 | 5 | 3 |
| NEO-FFI = NEO-Five Factor Inventory (111); PCL-R = Psychopathy Checklist-Revised (1); PPI-R = Psychopathic Personality Inventory-Revised (112); PSPS = Primary and Secondary Psychopathy Scales (113); SRP-SF = Self-Report Psychopathy-Short Form (114)  E = Empathy; FEP = Facial Emotion Processing; MJ = Moral Judgment; NC = Not Categorized  *Of the eleven studies that reported findings for the two clusters of psychopathic traits (interpersonal/affective and impulsive lifestyle/antisocial), five studies controlled for the other cluster of traits in statistical models. | | | | | | | | | |

| **Table S4.** Significant associations between brain function and psychopathy | | | | | | |
| --- | --- | --- | --- | --- | --- | --- |
| **Task** | **Region(s)** | **Hemi.** | **Direction** | **Peak MNI Coordinates**  **(x, y, z)** | **Size (Voxels)** | **Threshold** |
| **Total Psychopathy** | | | | | | |
| All Functional | Anterior Cingulate | L | - | -6, 32, 22 | 293 | Extent |
|  | Dorsomedial Prefrontal Cortex | L | + | -2, 56, 28 | 1 | Height |
|  | Dorsomedial Prefrontal Cortex | L/R | + | -2, 50, 34 | 347 | Extent |
|  | Dorsomedial Prefrontal Cortex | L/R | + | 2, 44, 32 | 69 | Height |
|  | Inferior Frontal Gyrus (Orb.) | R | + | 40, 40, -8 | 301 | Extent |
|  | Posterior Orbitofrontal Cortex | R | + | 26, 8, -16 | 12 | Height |
|  | Amygdala/  Temporal Pole | R | + | 30, 4, -20 | 399 | Extent |
|  | Hippocampus/  Parahippocampal Gyrus | R | + | 24, -36, 2 | 105 | Extent |
|  | Hippocampus/  Parahippocampal Gyrus | R | + | 30, -40, -4 | 337 | Height |
|  | Cerebellum/  Cuneus/  Inferior Occipital Cortex/  Posterior Cingulate/  Precuneus/  Superior Occipital Cortex | L/R | + | 12, -70, 16 | 4272 | Extent |
| Facial Emotion Processing | *No significant clusters* |  | - |  |  |  |
|  | *Too few studies* |  | + |  |  |  |
| Empathy | Superior Temporal Gyrus | R | - | 46, -42, 16 | 106 | Height |
|  | Middle Temporal Gyrus | R | - | 48, -54, 10 | 18 | Height |
|  | Dorsomedial Prefrontal Cortex | L/R | + | -2, 48, 32 | 370 | Height |
|  | Amygdala/  Temporal Pole | R | + | 30, 4, -20 | 325 | Height |
|  | Hippocampus/  Parahippocampal Gyrus | R | + | 30, -40, -4 | 337 | Height |
| Moral Judgment | *No significant clusters* |  | - |  |  |  |
|  | Middle Frontal Gyrus | R | + | 36, 38, 20 | 433 | Height |
| **Interpersonal/Affective Psychopathic Traits** | | | | | | |
| All Functional | *No significant clusters* |  | - |  |  |  |
|  | *No significant clusters* |  | + |  |  |  |
| Facial Emotion Processing | *Too few studies* |  | - |  |  |  |
|  | Dorsomedial Prefrontal Cortex | L/R | + | 0, 36, 40 | 515 | Height |
|  | Inferior Frontal Gyrus (Tri.)/  Middle Frontal Gyrus | R | + | 48, 34, 16 | 495 | Height |
|  | Inferior Frontal Gyrus (Op.)/  Rolandic Operculum | L | + | -44, 8, 14 | 485 | Height |
| Empathy | *No significant clusters* |  | - |  |  |  |
|  | *No significant clusters* |  | + |  |  |  |
| Moral Judgment | *No significant clusters* |  | - |  |  |  |
|  | *Too few studies* |  | + |  |  |  |
| **Impulsive Lifestyle/Antisocial Psychopathic Traits** | | | | | | |
| All Functional | *No significant clusters* |  | - |  |  |  |
|  | *No significant clusters* |  | + |  |  |  |
| Facial Emotion Processing | *Too few studies* |  | - |  |  |  |
|  | *Too few studies* |  | + |  |  |  |
| Empathy | *Too few studies* |  | - |  |  |  |
|  | *Too few studies* |  | + |  |  |  |
| Moral Judgment | *No significant clusters* |  | - |  |  |  |
|  | *Too few studies* |  | + |  |  |  |

| **Table S5.** Significant associations between brain structure and total psychopathy excluding studies of community samples | | | | | | |  |
| --- | --- | --- | --- | --- | --- | --- | --- |
| **Measure** | **Region(s)** | **Hemi.** | **Direction** | **Peak MNI Coordinates**  **(x, y, z)** | **Size**  **(Voxels)** | **Threshold** | |
| All Structural | *No significant clusters* |  | - |  |  |  | |
|  | Caudate/  Nucleus Accumbens | L | + | -10, 18, -4 | 112 | Height | |
| GMV | Dorsomedial Prefrontal Cortex/  Superior Frontal Gyrus | L | - | -12, 58, 20 | 674 | Extent | |
|  | Dorsomedial Prefrontal Cortex/  Superior Frontal Gyrus | R | - | 12, 44, 38 | 757 | Extent | |
|  | Fusiform Gyrus/  Inferior Temporal Gyrus/  Medial Temporal Pole | L | - | -34, -4, -40 | 759 | Extent | |
|  | Fusiform Gyrus/  Inferior Temporal Gyrus/  Medial Temporal Pole | R | - | 36, 6, -40 | 789 | Extent | |
|  | Caudate | L | + | -10, 18, -2 | 112 | Height | |
|  |  |  |  |  |  |  | |
|  |  |  |  |  |  |  | |

| **Table S6.** Significant associations between brain function and total psychopathy excluding studies of community samples | | | | | |
| --- | --- | --- | --- | --- | --- |
| **Region(s)** | **Hemi.** | **Direction** | **Peak MNI Coordinates**  **(x, y, z)** | **Size**  **(Voxels)** | **Threshold** |
| Temporoparietal Junction | R | - | 52, -54, 12 | 1 | Height |
| Temporoparietal Junction | R | - | 50, -54, 16 | 1 | Height |
| Dorsomedial Prefrontal Cortex | L | + | -2, 56, 28 | 1 | Height |
| Dorsomedial Prefrontal Cortex | L | + | -2, 50, 34 | 347 | Extent |
| Dorsomedial Prefrontal Cortex | L/R | + | 2, 44, 32 | 69 | Height |
| Posterior Orbitofrontal Cortex | R | + | 26, 8, -16 | 12 | Height |
| Amygdala/  Temporal Pole | R | + | 30, 4, -20 | 399 | Extent |
| Hippocampus/  Parahippocampal Gyrus | R | + | 24, -36, 2 | 105 | Extent |
| Hippocampus/  Parahippocampal Gyrus | R | + | 30, -40, -4 | 337 | Height |
| Cerebellum/  Cuneus/  Inferior Occipital Cortex/  Posterior Cingulate/  Precuneus/  Superior Occipital Cortex | L/R | + | 12, -70, 16 | 4563 | Extent |
|  |  |  |  |  |  |
